# Influence of non-pharmaceutical interventions during the COVID-19 pandemic on respiratory viral infections – a prospective population-based cohort study

**DOI:** 10.3389/fpubh.2024.1415778

**Published:** 2024-06-24

**Authors:** Nadja Käding, Frederike Waldeck, Bjarne Meier, Sébastien Boutin, Max Borsche, Alexander Balck, Bandik Föh, Jan Kramer, Christine Klein, Alexander Katalinic, Jan Rupp

**Affiliations:** ^1^Department of Infectious Diseases and Microbiology, University Hospital Schleswig-Holstein, Lübeck, Germany; ^2^Airway Research Center North (ARCN), Member of the German Center for Lung Research (DZL), Lübeck, Germany; ^3^Institute of Neurogenetics, University of Lübeck, Lübeck, Germany; ^4^Department of Medicine I, University Hospital Schleswig-Holstein, Lübeck, Germany; ^5^LADR Laboratory Group Dr. Kramer and Colleagues, Geesthacht, Germany; ^6^Institute of Social Medicine and Epidemiology, University of Lübeck, Lübeck, Germany

**Keywords:** non-pharmaceutical interventions, respiratory viruses, infection risk, virus distribution, behavioral factors

## Abstract

Non-pharmaceutical interventions (NPI) have been proven successful in a population-based approach to protect from SARS-CoV-2 transmission during the COVID-19 pandemic. As a consequential-effect, a reduction in the spread of all respiratory viruses has been observed, but the primary factors behind this phenomenon have yet to be identified. We conducted a subgroup analysis of participants from the ELISA study, a prospective longitudinal cohort study on SARS-CoV-2 transmission, at four timepoints from November 2020 – September 2022. The aim was to provide a detailed overview of the circulation of respiratory viruses over 2 years and to identify potential personal risk factors of virus distribution. All participants were screened using qPCR for respiratory viral infections from nasopharyngeal swabs and answered a questionnaire regarding behavioral factors. Several categories of risk factors for the transmission of respiratory viruses were evaluated using a scoring system. In total, 1,124 participants were included in the study, showing high adherence to governmental-introduced NPI. The overall number of respiratory virus infections was low (0–4.9% of participants), with adenovirus (1.7%), rhino−/enterovirus (3.2%) and SARS-CoV-2 (1.2%) being the most abundant. We detected an inverse correlation between the number and intensity of NPI and the number of detected respiratory viruses. More precisely, the attendance of social events and household size was associated with rhino−/enterovirus infection while social contacts were associated with being positive for any virus. NPI introduced during the COVID-19 pandemic reduced the occurrence of seasonal respiratory viruses in our study, showing different risk-factors for enhanced transmission between viruses.

**Trial registration:**

DRKS.de, German Clinical Trials Register (DRKS), Identifier: DRKS00023418, Registered on 28 October 2020.

## Introduction

With the emergence of the coronavirus disease 2019 (COVID-19) pandemic, non-pharmaceutical interventions (NPI) were introduced worldwide to limit SARS-CoV-2 transmission. NPI are pandemic countermeasures that are readily available at all times and in all countries (1). NPI can be roughly divided into interventions that reduce personal contacts (quarantine, isolation, cohorting, stay-at-home orders) and make contacts safer (physical distancing, hand hygiene, masks) (2). The efficacy of NPI on the evolution of pandemics must be weighed against the restriction in personal autonomy of decisions and high economic costs (3, 4). Only a few studies have evaluated the efficacy of NPI on the distribution of respiratory viruses before the COVID-19 pandemic. Since then a growing body of evidence shows the effect of NPI on SARS-CoV-2 transmission and other respiratory viruses (5). The effectiveness of the different NPI is an ongoing matter of debate. Since NPI were introduced in bundles during the COVID-19 pandemic the effect of each NPI is hard to establish: Meta-analysis suggests high influence on SARS-CoV-2 reproduction numbers due to school, workplace, business and venue closure and the ban of public events and intermediate effectiveness of lockdown, travel restrictions, ban of mass gathering and social events as well as public campaigns, wearing a mask and social distancing. Other NPI seems to be less effective including contact tracing and isolation/quarantine (6). Still, the effect of NPI on non-SARS-CoV-2 respiratory diseases has not been well studied thus far.

First restrictions on daily life were implemented in mid-March 2020 in the federal state of Schleswig-Holstein, Germany (7). These included the prohibition of private meetings and social events, and the closure of public institutions, stores, bars and restaurants; moreover, no tourism was allowed. In April 2020, face masks were introduced to reduce virus transmission to and from individuals (8). NPI were adapted throughout the pandemic according to SARS-CoV-2 circulation and the expected number of infected and/or hospitalized patients as well as mortality. NPI furthermore led to a markedly reduced transmission of respiratory viruses, including influenza virus and a strong disruption of the typical seasonal circulation patterns of common respiratory virus infections (9). The COVID-19 pandemic strongly influenced the typical flu season in winter (10), as significantly fewer infections were detected and seasonality of influenza virus infections was no longer observed. Lower numbers of infections with influenza viruses, rhinovirus, human metapneumoviruses, parainfluenza viruses and respiratory syncytial viruses have been detected in Germany since the beginning of the coronavirus pandemic 2020 according to the Robert Koch Institute, as well as reduced cases of acute respiratory infections (ARI) (10, 11).

ARI are a common cause of doctor’s consultation, hospitalization and death during the winter season. ARI are associated with high economic costs (550 Million euros within 2010–2019) (12). The annual incidence of ARI remains unclear, but it is estimated that most people suffer from at least one ARI per year (13). Surveillance performed in the United States of America reports yearly >25 million primary care and 9 million emergency department admissions due to ARI (14). Respiratory viruses may be responsible for almost 40% of CAP cases and an even higher proportion of ARIs (15). Therefore, prevention of ARI is a cost-effective and important public health measure.

This study aimed to investigate the impact of the COVID-19 pandemic protection measures including lockdown measures on the epidemiology of seasonal respiratory viruses, including influenza and SARS-CoV-2. We further aimed to describe predictors of virus distribution in Luebeck, Germany.

## Methods

### Study design and study procedures

We conducted a longitudinal, prospective cohort study, which was a sub-study of the Luebeck Longitudinal Investigation of SARS-CoV-2 Infection (ELISA) study. The methods have been described elsewhere (16, 17). In the original ELISA study protocol, the study visits included in our sub-study were previously described as study visit 6, 7 and two follow-ups but for clarity purpose they will be named here as study visit 1–4, respectively.

At each study visit we aimed for 500 participants. During study visit 1 (November 2020) and 2 (February 2021) study participants were randomly selected. While at study visit 3 (March 2022) and 4 (September 2022) study participants could enroll themselves to the sub-study, where we offered maximal 500 appointments. At study visit 2 we had to exclude 110 study participants, since they did not take part in the general testing intervals of the ELISA cohort. In total 1,124 study participants were tested via quantitative real-time polymerase chain reaction (PCR) for respiratory viruses from 1,879 nasopharyngeal swabs using the ampliCube Respiratory Viral Panel 1, 3, 4 and the ampliCube Coronavirus Panel Kits (MIKROGEN, Neuried) (16). Respiratory viruses included in the panels are influenza A/B, SARS-CoV-2, middle east respiratory syndrome coronavirus (MERS-CoV), human coronavirus (hCoV) (229E, HKU1, NL63, OC43), parechovirus, respiratory syncytial virus (RSV), metapneumovirus (HMPV), rhinovirus, enterovirus, and adenovirus.

For antibody testing, a venous blood sample was drawn. The follow-up tests were based on dried blood spots. For anti-nuclear capsid protein immunoglobulin G (anti-NCP IgG) testing, we performed an Anti-SARS-CoV-2-NCP-ELISA (IgG) (EUROIMMUN AG, Lübeck, Germany).

### Scoring system

Personal risk factors for the acquisition of respiratory viral infections were inquired via questionnaire. These included household size, social contacts, attendance and size of events, home office, use of public transportation and visit to physician’s office. A scoring system was developed based on the personal risk factors for viral transmission to calculate an overall risk for exposure to viral infections and to perform statistical analysis. Risk factors for viral transmission were weighted according to their reported efficacy in the protection of SARS-CoV-2 transmission, as has been reported in the introduction and in the literature including empirical studies (6, 18, 19). The risk score is shown in Supplementary Table 1. A maximum of 3 points could be achieved in each category. Self-reported greater size of the household and attended events became higher points on the scoring system since the limitation of event size has been reported to be associated with lower SARS-CoV-2 transmission (18). The category “social contacts” accounted for the cumulative risk of different social events including bars, theater and others. Visiting a bar, disco or restaurant was weighted higher than theater, hairdresser and gym, since there were no assigned/stationary seats or masks were not worn during eating and drinking (18).

### Statistics

Descriptive statistics and statistical analysis were performed using IBM SPSS Statistics 27.0 and R 4.2.2. *p*-values of <0.05 were considered statistically significant. To evaluate the association positivity of viral detection via PCR with the scoring system we analyzed the data in a multivariate logistic regression model (generalized linear model with binomial distribution) with age and gender as co-variates using the R package stats and the adjusted odds ratio (OR) was calculated from the model using the R package epiDisplay. Multi-collinearity was evaluated using Variance inflation factor (VIF) and spearman correlation. VIF were low to moderate (1.02–1.42) indicating low correlation but the spearman correlation indicates a strong association between three scores which were then included as interacting scores in the model (Supplementary Figure 1). To assess whether there is a correlation between the transmission of respiratory viruses and the intensity of NPI, we performed a multivariate analysis on the probability of positivity to any viruses as well as individual probability for the most prevalent viruses; RV/EV, adenovirus and SARS-CoV-2.

## Results

### Study demographics

The ELISA cohort consisted of 3,051 participants, representing ~1% of the local population in the Luebeck catchment area. Further, the cohort included a high-exposure subgroup to enrich potential positivity based on a profession requiring intense and/or frequent contact with other people, such as healthcare personnel. Study participants of the ELISA cohort had an above-average educational level (17). From the 1,124 participants tested, 517 people (45.9%) were tested once vs. 473 (42.1%) tested twice vs. 120 (10.7%) tested three times and 14 tested four times (1.3%). The median age of the ELISA cohort subgroup was 47 years (SD, 14.4; range, 18–79 years) and 54.5% were females, 13.8% active smokers, corresponding to the ELISA cohort. 56.5% of participants were vaccinated at least once against influenza and 17.2% against pneumococcus. COVID-19 vaccination status is shown in Supplementary Table 2 and corresponds to 0–100% vaccinated participants at timepoint 1–4.

### Regional COVID-19 NPIs and distribution of seasonal respiratory viruses

**Figure 1:**
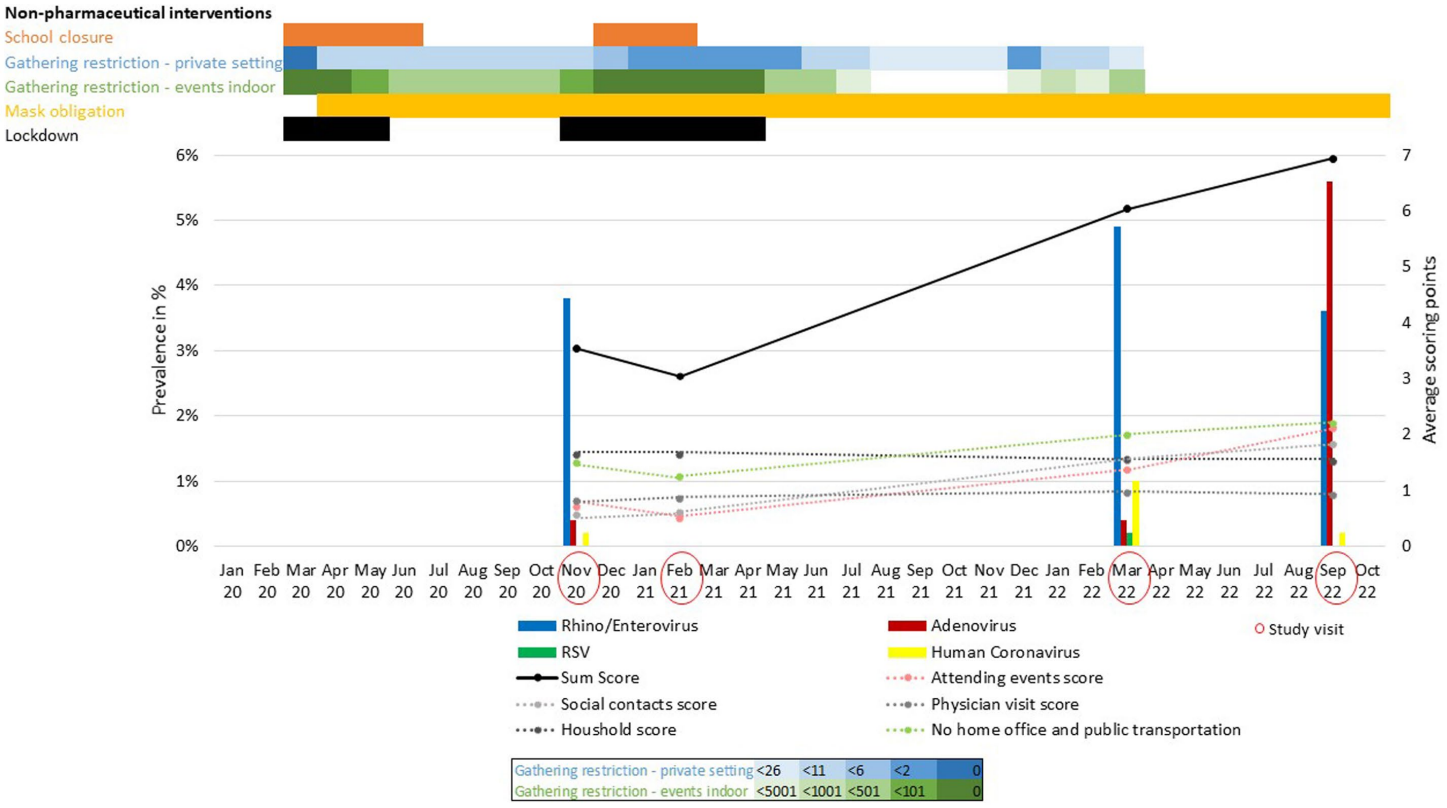
Regional governmental COVID-19 non-pharmaceutical interventions and prevalence of respiratory viruses during the COVID-19 pandemic. Intensity of non-pharmaceutical interventions (NPI) (Law and Ordinance Gazettes of the Government of Schleswig-Holstein, https://www.schleswig-holstein.de/DE/) is indicated by different color intensities. Total score (black line) and single parameters (dotted lines) of the scoring system indicate adherence to NPI during the study period.

During study visit 1 (November 2020) rhinovirus and enterovirus (RV/EV) (19 of 500, 3.8% positive) circulation could be observed after a period where restrictions were eased in the summer and early fall, while cases of human coronavirus, SARS-CoV-2, adenovirus and parechovirus could be infrequently detected (Figure 1 and Supplementary Table 2). No respiratory virus could be detected at visit 2 in February 2021 during the complete lockdown. At study visit 3 March 2022 we could observe an increase in RV/EV (24 of 493, 4.9%). During that time point SARS-CoV-2 was detected in 14 participants (2.8%) and human coronavirus in five cases (1%). Low numbers of adenovirus (2/493, 0.4%), RSV (1/493, 0.2%) and HMPV (2/493, 0.4%) could be detected (Supplementary Table 2). At study visit 4 in September 2022, when COVID-19 measures were not present anymore, adenoviruses (28/496, 5.6%), as well as RV/EV (18/496, 3.6%), could be detected in the study group. Only single cases of human coronavirus and SARS-CoV-2 with five cases could be detected (Supplementary Table 2), but no case of influenza. Overall, the most frequent viruses were RV/EV (61/1879, 3.2%) followed by adenovirus (32/1879, 1.7%).

### Predisposing personal risk factors of respiratory viral infection

**Figure 2:**
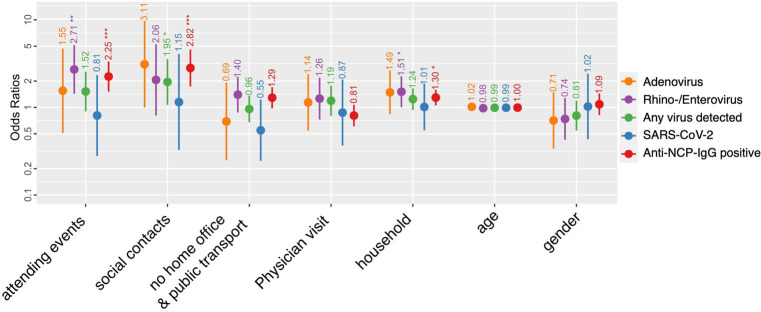
Predisposing factors of respiratory viral infection. Odds ratios and 95% confidence intervals for adenovirus, rhino−/enterovirus and SARS-CoV-2 infection as well as any virus detected and being exposed to SARS-CoV-2 [anti-nucleocapsid (NCP) antibodies positive] for selected parameters of interest. The antibody detection assay was performed as described previously (16). Only the additive factors are displayed but the interaction due to collinearity are shown in the Supplementary Figure 1B.

The multivariate analysis on the probability of positivity to any viruses as well as individual probability for RV/EV, adenovirus and SARS-CoV-2 is shown in Figure 2. The size of social events was associated with being positive for RV/EV (OR: 2.71, 95% CI: 1.43–5.14) and having had recent SARS-CoV-2 infection (positivity for anti-NCP IgG) (OR: 2.25, 95% CI 1.52–3.43). Social contacts were associated with being positive for any virus (OR: 1.95, 95% CI 1.07–3.56) and recent SARS-CoV-2 infection (OR: 2.82, 95% 1.73–4.58). The household size correlated with being RV/EV positive (OR: 1.51, 95% CI 1.01–2.26) and having anti-NCP IgG (OR: 1.3, 95% CI 1.06–1.59). No impact on virus transmission was detected by visiting a physician’s office, using public transportation and going to work.

The interaction of attending events and social contacts showed a significant association with recent SARS-CoV-2 infection. As well as the interaction of the factor social contacts and using public transportation and going to work. While the interaction of attending events and using public transportation and going to work correlate with being RV/EV positive.

## Discussion

Our data provide a detailed picture of the circulation of respiratory viruses in a population-based prospective study over 2 years during the COVID-19 pandemic. Because of the COVID-19 pandemic, several NPI were implemented by the federal government of Germany (Figure 1). In March 2020 a nationwide lockdown was introduced, followed by school closures, and very limited personal contacts (Figure 1). Further, mask obligation was introduced and lasted for the complete study period. While surveillance tools for respiratory viruses report incidences of symptomatic patients with acute respiratory infections that approach doctor’s offices, our data characterize the “real world” circulation of respiratory viruses in the population. Therefore, the effect of NPI on respiratory virus transmission in the Luebeck area determined by our study can be conveyed to the local population. To the best of our knowledge, this is the first study to show a correlation between specific personal risk factors, including size of household and attended events, and the prevalence of non-SARS-CoV-2 respiratory viruses in the general population.

The inverse correlation between the number and intensity of NPI and the number of detected respiratory viruses, including adenovirus and RV/EV, suggests an influence of NPI on respiratory virus transmission other than SARS-CoV-2. The effect of the bundled NPI (e.g., social distancing, travel ban, lockdown, isolation/quarantine, personal protective equipment, school and workplace closure) on the reduction of SARS-CoV-2 transmission has been established (20). Their impact might be most pronounced and might only work in bundles (21).

Surprisingly, few studies evaluated the effect of NPI on seasonal respiratory viruses. NPI work through the reduction of personal contacts and enforcement of hygiene measures. Most respiratory viruses are transmitted by droplets (9), therefore an impact of COVID-19 NPI was to be expected. Usually, RV circulate all year round, but are reduced during winter season. In the season 2018/19 highest positivity rates for influenza were recorded from January until mid of March. RSV circulates from November through March with a peak at the end of year, while HMPV occurs all year round with higher activity in spring and summer (10, 22). Epidemiological data show a decrease in influenza circulation and an interruption of seasonal circulation of respiratory viruses during the pandemic, which suggests an effect of NPI on respiratory viruses other than SARS-CoV-2 (9). This could be confirmed by our cohort. No respiratory virus could be detected during the complete lockdown with school closures and strong restrictions regarding personal contacts (Figure 1). While total virus numbers partially increased when gathering restrictions were only limited to 25 people and indoor events allowed up to 500 people in the Luebeck area. A decrease has been shown for adenovirus, RSV, human coronavirus, metapneumovirus and influenza (4, 9) and resulted in decreased mortality and hospitalization rates of non-SARS-CoV-2 respiratory viruses (9). Other studies show a weaker effect of NPI on non-enveloped viruses, including adenovirus, bocavirus and RV (23). RV prevalence has been promoted as an indicator of the efficacy of measures against SARS-CoV-2 (24). RV is the most prevalent respiratory virus in humans and is widely distributed in the community (25). It has low seasonality and a similar transmission route as SARS-CoV-2. RV has been shown to respond quickly to anti-COVID-19 measures (26). Our data further stress the importance of reducing social events and contacts to contain respiratory viruses in pandemics. Furthermore, the interaction of NPI (social contact, attending events and public transport/attending work in particular) were associated with spread of SARS-CoV-2 and RV/EV which adds important knowledge for the preparedness to future pandemics. Even though this study was limited to the Luebeck area, similar NPI and a decrease in respiratory virus detection has also been shown for other German regions (21, 23).

RV/EV and adenovirus were the most abundant respiratory viruses, especially in the summer of 2022 in the Luebeck area, when COVID-19 NPI were not present anymore. RV/EV and adenovirus are characterized by the absence of a viral envelope. The absence of a viral envelope may allow infections to quickly increase when infection control measures are relaxed because of increased stability on surfaces (9). Also, adenovirus and RV can be transmitted not only by droplets, but also by direct contact. They are shed from infected vectors for up to 3 weeks, have long stability in the environment and are resistant to disinfectants (5). This might explain why - in contrast to other respiratory viruses which are transmitted via droplet – RV/EV were significantly associated with household and attending events. Therefore, our score differentiates the effect of NPI according to the characteristics of specific respiratory viruses. Studies observed a viral interference between RV and influenza that led to a reduction in influenza infection (22, 27). Previous infection with RV inhibits infection with the influenza A virus by activating antiviral defenses in the target tissue of both viruses (27). At the same time, prior infection with influenza, in turn, may inhibit RV replication (28).

Our data were limited by the low prevalence of viruses and the high adherence of our cohort to governmental-introduced NPIs which could be shown by low total scores, especially during study visit 1 and 2. The recruitment process which tends to include healthy participants which willingly come to the study center might have led to an underestimation of respiratory viruses in the general population. Further, attrition remains a disadvantage of longitudinal studies. Interestingly, with growing rates of vaccinated participants adherence to NPI decreased resulting in higher respiratory virus circulation in the study population. We cannot differentiate the influence of extenuated NPIs from the effect of increasing SARS-CoV-2 vaccination status of participants on respiratory virus circulation. Studies suggest that influenza and pneumococcal vaccination are associated with a reduced risk of SARS-CoV-2 infection (29). Influenza vaccination is associated with reduced risk of ARI in adults (30, 31) and hospitalization due to RSV in children (32). To our knowledge no studies have evaluated the effect of SARS-CoV-2 vaccination on other respiratory viruses. This analysis might be hampered due to NPI which were adapted at the same time as vaccination coverage rate increased during COVID-19 pandemic. Our study is limited to the Luebeck area but markedly decreased rates of respiratory virus circulation have been shown for other German regions which might confirm generalizability of our results (21, 23).

In conclusion, we demonstrate that COVID-19 pandemic protection measures reduced the occurrence of seasonal respiratory viruses in the Luebeck area. Based on the proposed calculated score, we could define risk factors for virus transmission, which can be targeted measures for upcoming pandemics to reduce virus transmission.

## Data availability statement

The original contributions presented in the study are included in the article/Supplementary material, further inquiries can be directed to the corresponding author.

## Ethics statement

The studies involving humans were approved by Ethics Committee of the University of Luebeck. The studies were conducted in accordance with the local legislation and institutional requirements. The participants provided their written informed consent to participate in this study.

## Author contributions

NK: Writing – original draft, Writing – review & editing, Conceptualization, Investigation. FW: Writing – original draft, Writing – review & editing, Conceptualization, Investigation. BM: Formal analysis, Investigation, Writing – review & editing. SB: Formal analysis, Writing – review & editing. MB: Conceptualization, Investigation, Writing – review & editing. AB: Conceptualization, Investigation, Writing – review & editing. BF: Conceptualization, Investigation, Writing – review & editing. JK: Methodology, Resources, Writing – review & editing. CK: Conceptualization, Funding acquisition, Project administration, Supervision, Writing – review & editing. AK: Conceptualization, Funding acquisition, Project administration, Supervision, Writing – review & editing. JR: Conceptualization, Funding acquisition, Project administration, Supervision, Writing – review & editing.
