# Supplementary material for: Influence of non-pharmaceutical interventions during the COVID-19 pandemic on respiratory viral infections – a prospective population-based cohort study
Source: Front Public Health. 2024 Jun 24;12:1415778. doi: 10.3389/fpubh.2024.1415778 (PMC11228307; doi:10.3389/fpubh.2024.1415778)
Supplement: Supplementary file 1 [file Presentation_1.PPTX]

## Slide 1
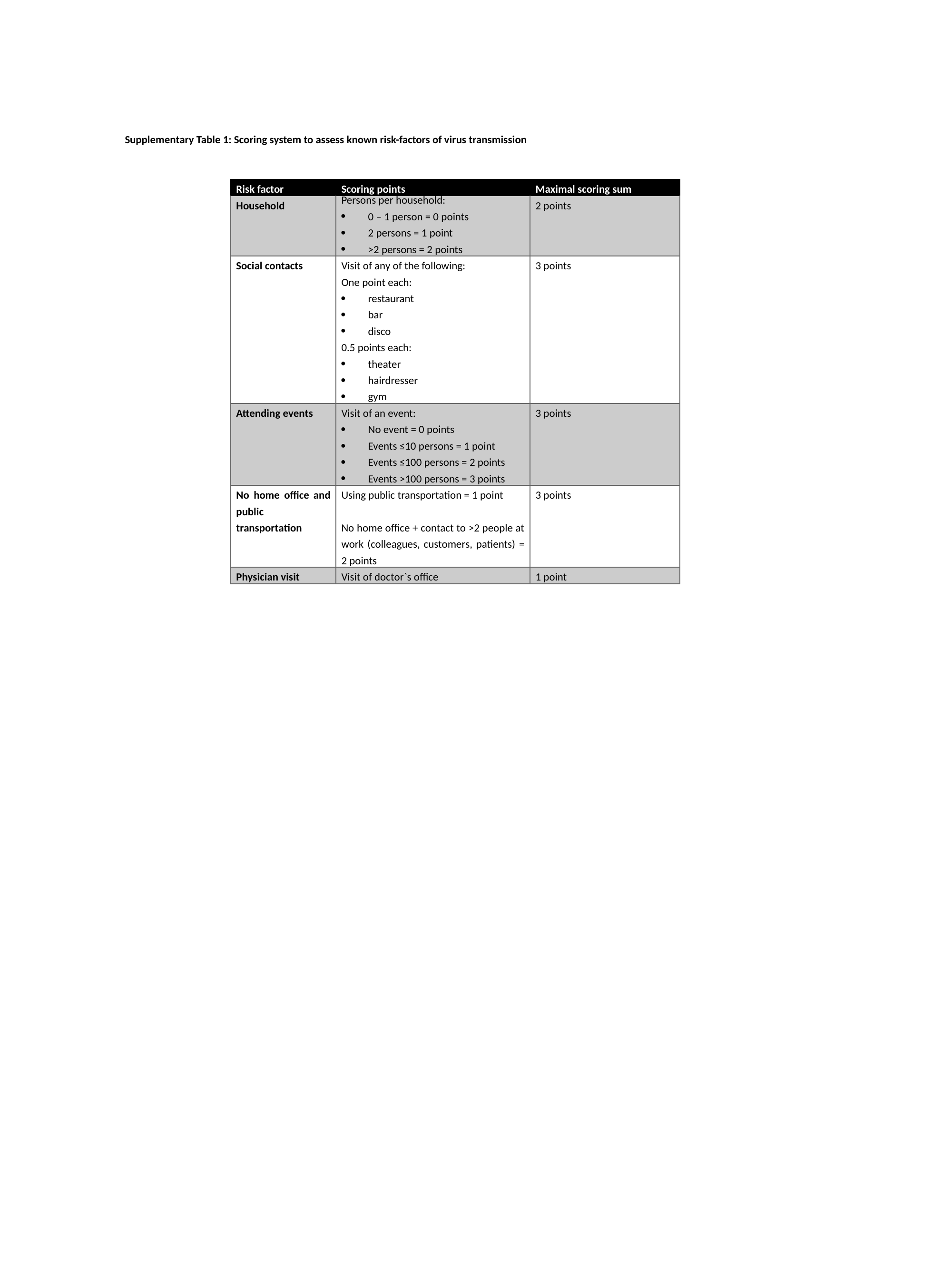

Supplementary Table 1: Scoring system to assess known risk-factors of virus transmission
| Risk factor | Scoring points | Maximal scoring sum |
| --- | --- | --- |
| Household | Persons per household: 0 – 1 person = 0 points 2 persons = 1 point >2 persons = 2 points | 2 points |
| Social contacts | Visit of any of the following: One point each: restaurant bar disco 0.5 points each: theater hairdresser gym | 3 points |
| Attending events | Visit of an event: No event = 0 points Events ≤10 persons = 1 point Events ≤100 persons = 2 points Events >100 persons = 3 points | 3 points |
| No home office and public transportation | Using public transportation = 1 point   No home office + contact to >2 people at work (colleagues, customers, patients) = 2 points | 3 points |
| Physician visit | Visit of doctor`s office | 1 point |
