# Supplementary material for: Influence of non-pharmaceutical interventions during the COVID-19 pandemic on respiratory viral infections – a prospective population-based cohort study
Source: Front Public Health. 2024 Jun 24;12:1415778. doi: 10.3389/fpubh.2024.1415778 (PMC11228307; doi:10.3389/fpubh.2024.1415778)
Supplement: Supplementary file 2 [file Presentation_2.PPTX]

## Slide 1
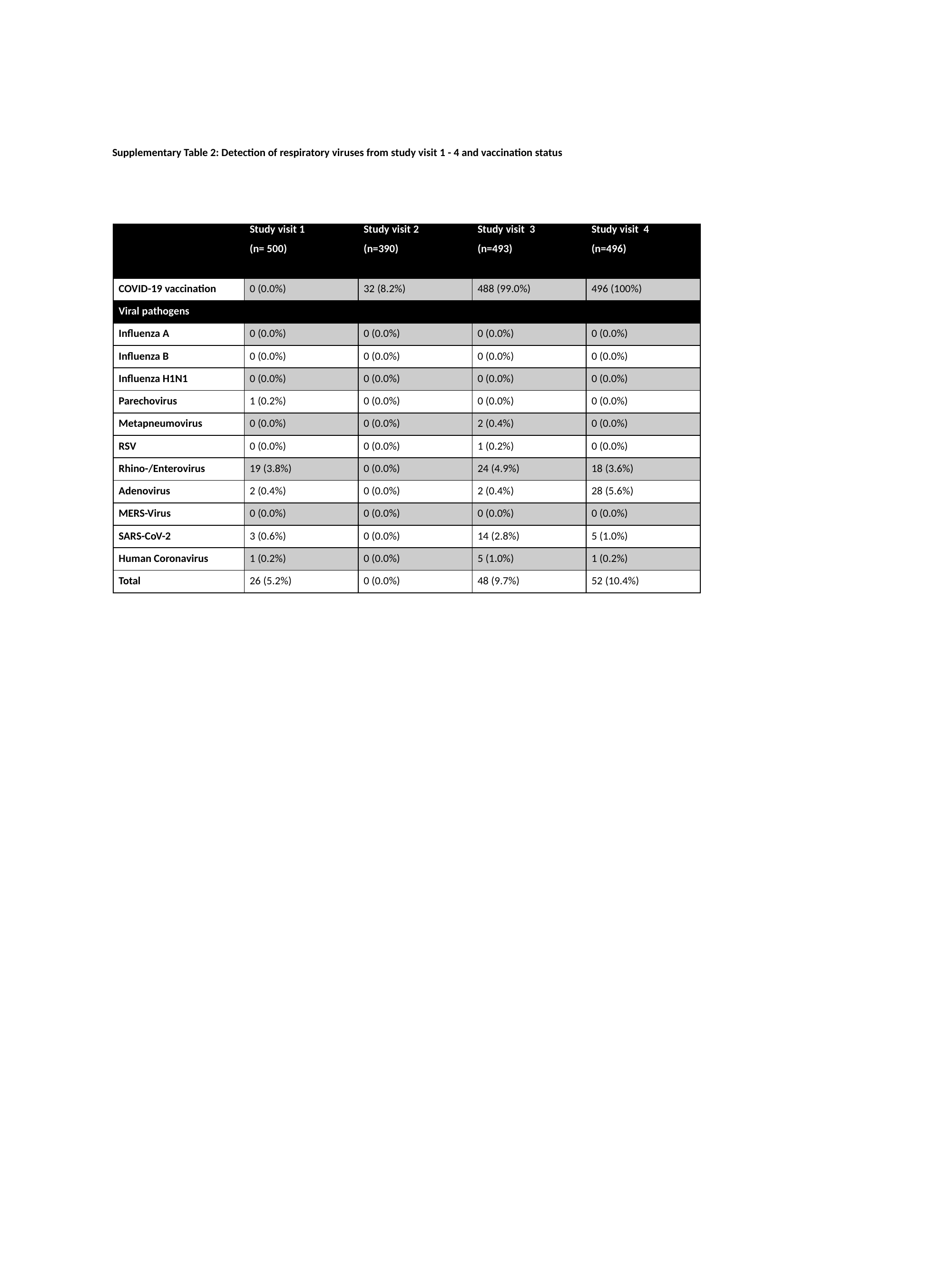

Supplementary Table 2: Detection of respiratory viruses from study visit 1 - 4 and vaccination status
| | Study visit 1 (n= 500) | Study visit 2 (n=390) | Study visit 3 (n=493) | Study visit 4 (n=496) |
| --- | --- | --- | --- | --- |
| COVID-19 vaccination | 0 (0.0%) | 32 (8.2%) | 488 (99.0%) | 496 (100%) |
| Viral pathogens | | | | |
| Influenza A | 0 (0.0%) | 0 (0.0%) | 0 (0.0%) | 0 (0.0%) |
| Influenza B | 0 (0.0%) | 0 (0.0%) | 0 (0.0%) | 0 (0.0%) |
| Influenza H1N1 | 0 (0.0%) | 0 (0.0%) | 0 (0.0%) | 0 (0.0%) |
| Parechovirus | 1 (0.2%) | 0 (0.0%) | 0 (0.0%) | 0 (0.0%) |
| Metapneumovirus | 0 (0.0%) | 0 (0.0%) | 2 (0.4%) | 0 (0.0%) |
| RSV | 0 (0.0%) | 0 (0.0%) | 1 (0.2%) | 0 (0.0%) |
| Rhino-/Enterovirus | 19 (3.8%) | 0 (0.0%) | 24 (4.9%) | 18 (3.6%) |
| Adenovirus | 2 (0.4%) | 0 (0.0%) | 2 (0.4%) | 28 (5.6%) |
| MERS-Virus | 0 (0.0%) | 0 (0.0%) | 0 (0.0%) | 0 (0.0%) |
| SARS-CoV-2 | 3 (0.6%) | 0 (0.0%) | 14 (2.8%) | 5 (1.0%) |
| Human Coronavirus | 1 (0.2%) | 0 (0.0%) | 5 (1.0%) | 1 (0.2%) |
| Total | 26 (5.2%) | 0 (0.0%) | 48 (9.7%) | 52 (10.4%) |
