# Supplementary material for: Influence of non-pharmaceutical interventions during the COVID-19 pandemic on respiratory viral infections – a prospective population-based cohort study
Source: Front Public Health. 2024 Jun 24;12:1415778. doi: 10.3389/fpubh.2024.1415778 (PMC11228307; doi:10.3389/fpubh.2024.1415778)
Supplement: Supplementary file 3 [file Presentation_3.PPTX]

## Slide 1
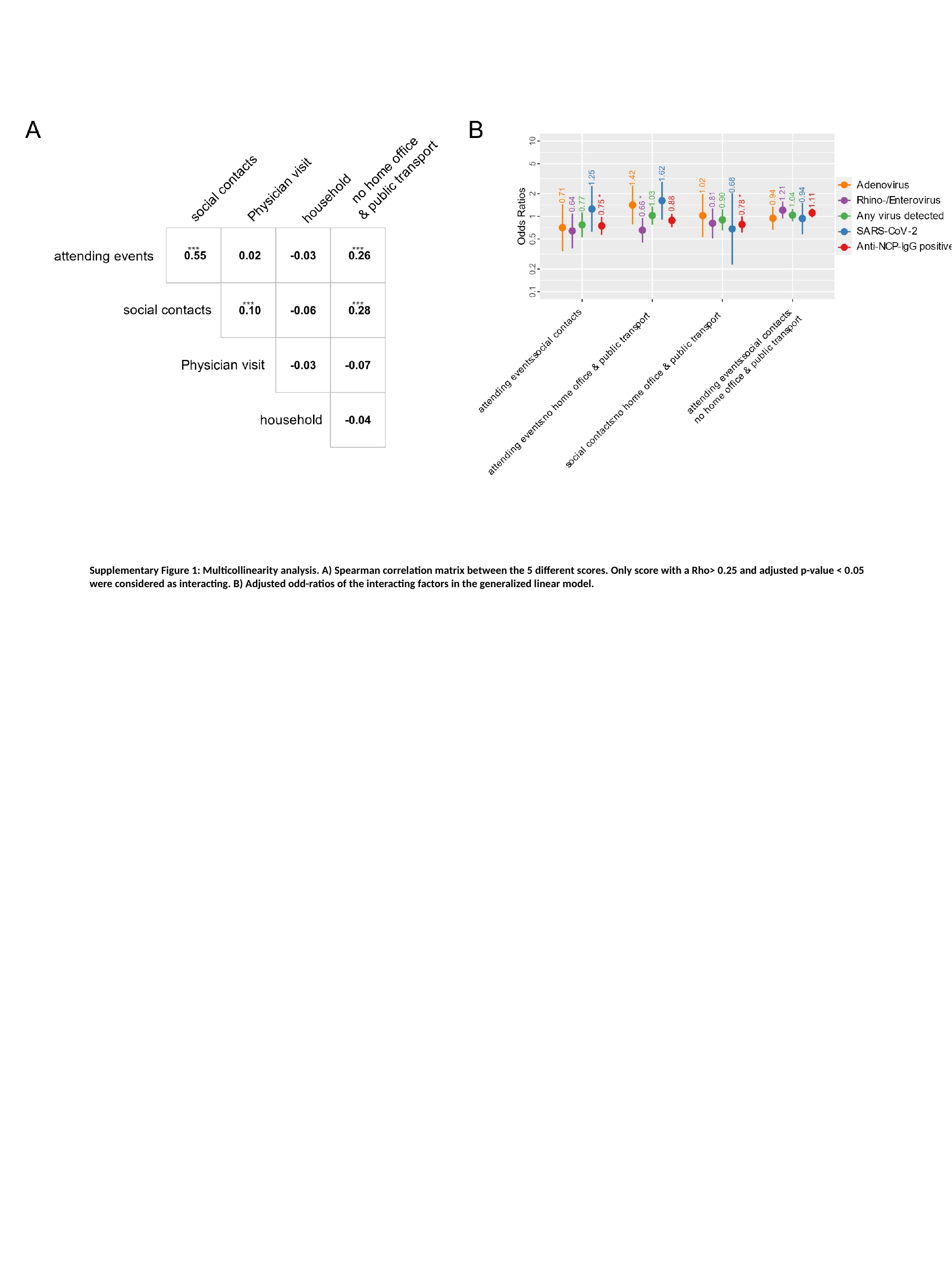

Supplementary Figure 1: Multicollinearity analysis. A) Spearman correlation matrix between the 5 different scores. Only score with a Rho> 0.25 and adjusted p-value < 0.05 were considered as interacting. B) Adjusted odd-ratios of the interacting factors in the generalized linear model.
